# Supplementary material for: Examining the Role of Traditional Masculinity and Depression in Men’s Risk for Contracting COVID-19
Source: Behav Sci (Basel). 2022 Mar 16;12(3):80. doi: 10.3390/bs12030080 (PMC8945728; doi:10.3390/bs12030080)
Supplement: Supplementary file 1 [file behavsci-12-00080-s001.zip › behavsci-1509183-supplementary.pdf]

Examining the role of traditional masculinity and depression in men's risk for contracting COVID-19

Walther, A.[1,*], Eggenberger, L.[1], Grub, J.[1], Ogrodniczuk, J.S.[2], Seidler, Z.E.[3,4], Rice, S.M.[3,4], Kealy, D.[2], Oliffe, J.L.[5], Ehlert, U.[1]

[1] Clinical Psychology and Psychotherapy, University of Zurich, Zurich, Switzerland
[2] Department of Psychiatry, University of British Columbia, Vancouver, Canada
[3] Centre for Youth Mental Health, The University of Melbourne, Parkville, VIC, Australia
[4] Orygen, Melbourne, VIC, Australia
[5] School of Nursing, University of British Columbia, Vancouver, Canada
[*] Corresponding author

Supplementary Material

Figure S1. *Empirical and Theoretical Normal Cumulative Distribution Functions (CDF) of the Study Variables* page 1

Figure S2. *Standardized Odds Ratios for COVID-19 Infection and their two- and one-sided 95% Confidence Intervals* page 2

Table S1. *Results of Mean Score Comparisons between Men with and without COVID-19 Infection* page 3

Table S2. *Correlation Matrix for Study Variables Including Subscales* page 4

Table S3. *Results of Binary Logistic Regression Analyses predicting COVID-19 Infection using Total Scores* page 5

Table S4. *Results of Binary Logistic Regression Analyses predicting COVID-19 Infection using Subscales* page 6

Table S5. *Results of Univariate, Binary Logistic Regression Analyses predicting COVID-19 Infection* page 7

Table S6. *Variance Inflation Factors (VIF) for the Logistic Regression Models* page 8

S-Discussion: *Discussion part on traditional masculinity and specific COVID-19-related symptoms* page 9

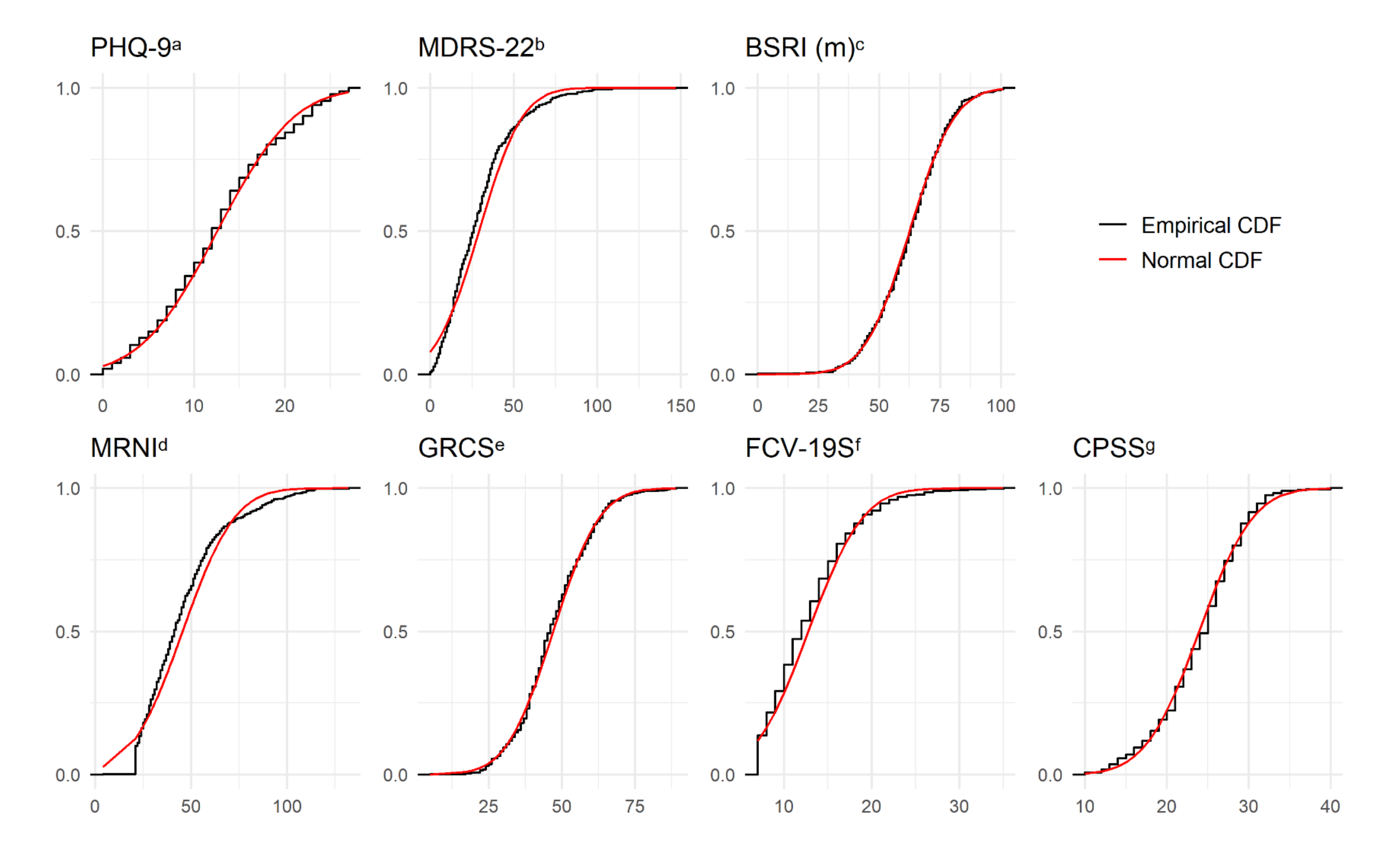

Figure S1. *Empirical and Theoretical Normal Cumulative Distribution Functions (CDF) of the Study Variables*

Note. [a] PHQ-9 = Patient health Questionnaire – 9; [b] MDRS-22 = Male Depression Risk Scale – 22; [c] BSRI (m) = Bem Sex-Role Inventory (masculinity subscale); [d] MRNI = Male Role Norms Inventory; [e] GRCS = Gender Role Conflict Scale; [f] FCV-19S = Fear of COVID-19 Scale; [g] CPSS = COVID-19 Pandemic Stress Scale.

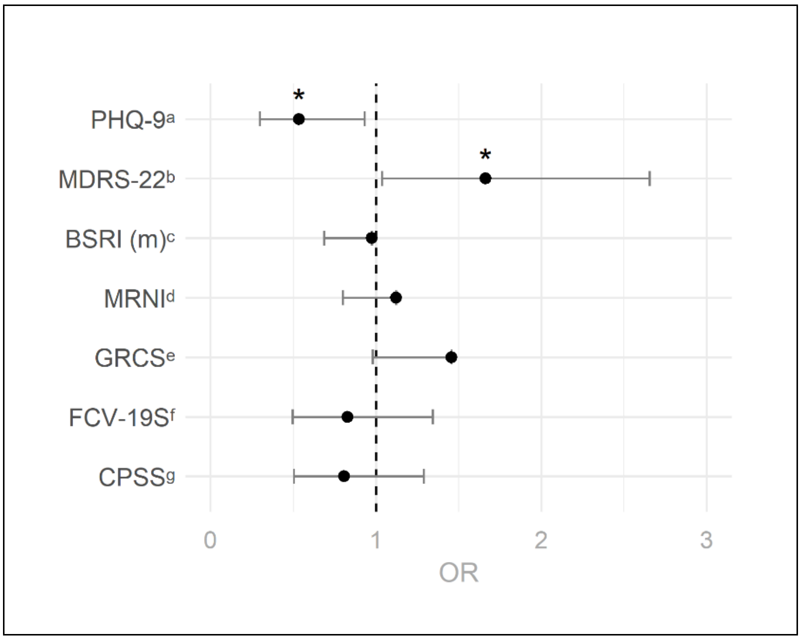

Figure S2. *Standardized Odds Ratios for COVID-19 Infection and their two- and one-sided 95% Confidence Intervals*

Note. OR = Odds ratio
[a] PHQ-9 = Patient health Questionnaire – 9; [b] MDRS-22 = Male Depression Risk Scale – 22; [c] BSRI (m) = Bem Sex-Role Inventory (masculinity subscale); [d] MRNI = Male Role Norms Inventory; [e] GRCS = Gender Role Conflict Scale ; [f] FCV-19S = Fear of COVID-19 Scale; [g] CPSS = COVID-19 Pandemic Stress Scale.
[*] = $p < .05$

Table S1. *Results of Mean Score Comparisons between Men with and without COVID-19 Infection*

**Variable**	**No CV19 (*N* = 456)**	**CV19 (*N* = 34)**	***t (df)***	***p***	***p* (corr.)**	***d***
	M *(SD)*	M *(SD)*				
**PHQ-9**[a]	12.7 *(6.6)*	11.3 *(6.3)*	1.21 *(488)*	.225	.812	-.22
**MDRS-22**[b]	28.8 *(20.3)*	34.1 *(24.4)*	-1.45 *(488)*	.169	.812	.26
**BSRI (m)**[c]	62.2 *(14.6)*	65.4 *(15.2)*	-1.20 *(488)*	.116	.812	.21
**MRNI**[d] **(tot.)**	44.9 *(20.7)*	54.4 *(28.9)*	-1.87 *(36)*	**.025***	.408	.44
(NT)	4.3 *(3.1)*	5.6 *(4.8)*	-1.61 *(35)*	.054	.630	.41
(RE)	6.7 *(4.0)*	8.3 *(4.7)*	-2.12 *(488)*	**.024***	.408	.38
(SR)	10.5 *(4.9)*	11.6 *(5.4)*	-1.31 *(488)*	.100	.800	.23
(AF)	5.2 *(3.7)*	6.5 *(5.7)*	-1.27 *(35)*	.082	.760	.33
(IS)	4.7 *(3.1)*	5.7 *(3.9)*	-1.73 *(488)*	**.046***	.630	.31
(D)	4.4 *(2.9)*	6.1 *(5.0)*	-1.94 *(35)*	**.012***	.216	.55
(T)	9.1 *(5.0)*	10.6 *(5.7)*	-1.66 *(488)*	**.046***	.630	.30
**GRCS**[e] **(tot.)**	46.7 *(12.2)*	51.7 *(16.0)*	-1.78 *(36)*	**.042***	.630	.40
(SPC)	13.1 *(4.8)*	14.9 *(6.0)*	-1.75 *(36)*	**.044***	.630	.38
(RE)	12.5 *(5.6)*	13.9 *(5.2)*	-1.43 *(488)*	.076	.760	.26
(RABBM)	7.7 *(3.7)*	8.7 *(5.0)*	-1.12 *(36)*	.136	.812	.26
(CBWFR)	13.4 *(5.2)*	14.1 *(6.3)*	-0.80 *(488)*	.212	.812	.14
**FCV-19S**[f]	12.9 *(4.9)*	11.8 *(4.6)*	1.28 *(488)*	.200	.812	-.23
**CPSS**[g]	24.0 *(5.2)*	22.6 *(5.4)*	1.52 *(488)*	.130	.812	-.27

Note. One-sided *t*-tests were used for the BSRI (m), MRNI (including all its subscales), and GRCS (including all its subscales). *P*-values for the MDRS-22 as well as the MRNI and all its subscales were calculated by bootstrapping with 5000 repetitions. *M* = mean score, *SD* = standard deviation, *t* = *t*-value, *df* = degrees of freedom, *p* = *p*-value, *corr.* = corrected for multiple testing using the Holm-Bonferroni method, *d* = Cohen's *d*.
[a] PHQ-9 = Patient Health Questionnaire – 9; [b] MDRS-22 = Male Depression Risk Scale – 22; [c] BSRI (m) = Bem Sex-Role Inventory (masculinity subscale); [d] MRNI = Male Role Norms Inventory (subscales: NT = Negativity toward Sexual Minorities; RE = Restrictive Emotionality; SR = Self-reliance through Mechanical Skills; AF = Avoidance of Femininity; IS = Importance of Sex; D = Dominance; T = Toughness); [e] GRCS = Gender Role Conflict Scale (subscales: SPC = Success, Power, Competition; RE = Restrictive Emotionality; RABBM = Restrictive Affectionate Behavior Between Men; CBWFR = Conflicts Between Work and Leisure - Family Relations); [f] FCV-19S = Fear of COVID-19 Scale; [g] CPSS = COVID-19 Pandemic Stress Scale.
* = $p < .05$

Table S2. *Correlation Matrix for Study Variables Including Subscales*

	M	SD	1	2	3	4	4.1	4.2	4.3	4.4	4.5	4.6	4.7	5	5.1	5.2	5.3	5.3	6	7
1. PHQ-9[a]	12.6	6.6	–																	
2. MDRS-22[b]	29.2	20.6	**.64*****	–																
3. BSRI (m)[c]	62.5	14.6	**-.36*****	-.10	–															
4. MRNI[d]	45.6	21.4	-.06	**.17***	**.33*****	–														
4.1 NT	4.3	3.3	-.09	.09	**.27*****	**.70*****	–													
4.2 RE	6.8	4.1	-.01	**.19****	**.20*****	**.80*****	**.46*****	–												
4.3 SR	10.6	4.9	-.05	.08	**.25*****	**.73*****	**.36*****	**.49*****	–											
4.4 AF	5.3	3.9	-.09	.15	**.30*****	**.84*****	**.62*****	**.62*****	**.44*****	–										
4.5 IS	4.8	3.2	-.05	.08	**.22*****	**.71*****	**.43*****	**.45*****	**.40*****	**.65*****	–									
4.6 D	4.5	3.1	0	**.19****	**.25*****	**.82*****	**.70*****	**.61*****	**.40*****	**.74*****	**.61*****	–								
4.7 T	9.2	5.0	-.05	.14	**.30*****	**.83*****	**.41*****	**.67*****	**.65*****	**.59*****	**.50*****	**.55*****	–							
5. GRCS[e]	47.0	12.6	**.41*****	**.48*****	.01	**.39*****	**.23*****	**.44*****	**.24*****	**.34*****	**.24*****	**.32*****	**.33*****	–						
5.1 SPC	13.2	4.9	.12	**.22*****	**.28*****	**.41*****	**.20*****	**.36*****	**.29*****	**.35*****	**.28*****	**.34*****	**.40*****	**.65*****	–					
5.2 RE	12.6	5.6	**.38*****	**.39*****	**-.17***	.14	.04	**.29*****	.04	.11	.03	.08	.13	**.69*****	**.19****	–				
5.3 RABBM	7.8	3.8	.13	**.22*****	-.01	**.43*****	**.39*****	**.43*****	**.23*****	**.39*****	**.28*****	**.38*****	**.29*****	**.62*****	**.30*****	**.37*****	–			
5.4 CBWFR	13.4	5.3	**.35*****	**.36*****	-.05	.10	.03	.09	.09	.09	.08	.08	.07	**.60*****	**.20*****	**.16***	.10	–		
6. FCV-19S[f]	12.8	4.9	**.29*****	**.28*****	**-.16***	-.15	-.14	-.08	-.13	-.12	-.10	-.10	-.14	**.18****	.01	.07	.04	**.31*****	–	
7. CPSS[g]	23.9	5.2	**.31*****	**.28*****	.03	-.04	-.04	-.04	.01	-.02	-.07	-.04	-.04	**.24*****	.11	.08	.05	**.35*****	**.58*****	–

Note. M = mean, *SD* = standard deviation. *p*-values were adjusted for multiple testing using the Holm-Bonferroni method.

[a] PHQ-9 = Patient Health Questionnaire – 9; [b] MDRS-22 = Male Depression Risk Scale – 22; [c] BSRI (m) = Bem Sex-Role Inventory (masculinity subscale); [d] MRNI = Male Role Norms Inventory (NT = Negativity Towards Sexual Minorities, RE = Restrictive Emotionality, SR = Self-reliance through Mechanical Skills, AF = Avoidance of Femininity, IS = Importance of Sex, D = Dominance, T = Toughness); [e] GRCS = Gender Role Conflict Scale (SPC = Success/Power/Competition, RE = Restrictive Emotionality, RABBM Restrictive Affectionate Behavior Between Men, CBWFR = Conflict Between Work and Family Relations); [f] FCV-19S = Fear of COVID-19 Scale; [g] CPSS = COVID-19 Pandemic Stress Scale.

* = $p < .05$, ** = $p < .01$, *** = $p < .001$

Table S3. *Results of Binary Logistic Regression Analyses predicting COVID-19 Infection using Total Scores*

**Variable**	**β[1] *(SE)*[1]**	**OR[1]**	**95% CI**	***p***	***p (corr.)***
**Intercept**	-2.94 *(0.23)*	0.05	[0.03, 0.08]	< **.001*****	< **.001*****
**PHQ-9[a]**	-0.63 *(0.29)*	0.53	[0.30, 0.93]	**.031***	.338
**MDRS-22[b]**	0.51 *(0.24)*	1.66	[1.04, 2.65]	**.032***	.338
**BSRI (m)[c]**	-0.03 *(0.22)*	0.97	[0.69, ____[	.395	1
**MRNI[d]**	0.12 *(0.20)*	1.12	[0.80, ____[	.451	1
**GRCS[e]**	0.38 *(0.24)*	1.46	[0.98, ____[	.285	.479
**FCV-19S[f]**	-0.19 *(0.25)*	0.83	[0.49, 1.34]	.460	1
**CPSS[g]**	-0.21 *(0.24)*	0.81	[0.50, 1.29]	.369	1
**Covariates**					
Age	-0.21 *(0.21)*	0.81	[0.51, 1.18]	.318	1
Income	-0.04 *(0.21)*	0.96	[0.46, 1.25]	.860	1
Education	0.61 *(0.28)*	1.84	[1.12, 3.40]	**.031***	.338
Sexual Orientation	0.01 *(0.22)*	1.01	[0.63, 1.50]	.944	1
**Goodness of Fit**					
Information criteria	AIC = 247.3;	BIC = 297.7;	R^2 = 11.9 %		
Likelihood-ratio test	χ^2 = 23.7;	*df* = 11;	*p* = .015 *		

Note. [1] Coefficients *z*-standardized. One-sided *z*-tests were used for the BSRI (m), MRNI (including all its subscales), and GRCS (including all its subscales). *β* = estimate, *SE* = standard error, *CI = confidence interval*, *p* = *p*-value, *corr.* = corrected for multiple testing using the Holm-Bonferroni method, R^2 = Nagelkerke pseudo *R*-squared.
[a] PHQ-9 = Patient Health Questionnaire – 9; [b] MDRS-22 = Male Depression Risk Scale – 22; [c] BSRI (m) = Bem Sex-Role Inventory (masculinity subscale); [d] MRNI = Male Role Norms Inventory; [e] GRCS = Gender Role Conflict Scale; [f] FCV-19S = Fear of COVID-19 Scale; [g] CPSS = COVID-19 Pandemic Stress Scale.
* = *p* < .05, *** = *p* < .001

Table S4. *Results of Binary Logistic Regression Analyses predicting COVID-19 Infection using Subscales*

**Variable**	**β[1] *(SE)*[1]**	**OR[1]**	**95% CI**	***p***	***p (corr.)***
**Intercept**	-2.99 *(0.24)*	0.05	[0.03, 0.08]	< **.001*****	< **.001** ***
**PHQ-9[a]**	-0.75 *(0.31)*	0.47	[0.25, 0.85]	**.015***	.299
**MDRS-22[b]**	0.50 *(0.24)*	1.64	[1.01, 2.66]	**.043***	.766
**BSRI (m)[c]**	-0.06 *(0.23)*	0.94	[0.64, ____[	.395	1
**MRNI[d] subscales**					
(NT)	0.16 *(0.23)*	1.17	[0.79, ____[	.245	1
(RE)	0.02 *(0.29)*	1.02	[0.63, ____[	.471	1
(SR)	0.07 *(0.26)*	1.08	[0.69, ____[	.390	1
(AF)	-0.36 *(0.29)*	0.70	[0.43, ____[	.106	1
(IS)	0.01 *(0.24)*	1.01	[0.66, ____[	.478	1
(D)	0.38 *(0.27)*	1.46	[0.93, ____[	.081	1
(T)	-0.12 *(0.32)*	0.88	[0.52, ____[	.350	1
**GRCS[e] subscales**					
(SPC)	0.18 *(0.23)*	1.20	[0.82, ____[	.214	1
(RE)	0.32 *(0.23)*	1.38	[0.95, ____[	.078	1
(RABBM)	-0.04 *(0.23)*	0.96	[0.65, ____[	.421	1
(CBWFR)	0.20 *(0.22)*	1.22	[0.85, ____[	.179	1
**FCV-19S[f]**	-0.23 *(0.26)*	0.80	[0.46, 1.32]	.393	1
**CPSS[g]**	-0.15 *(0.25)*	0.86	[0.52, 1.40]	.535	1
**Covariates**					
Age	-0.23 *(0.23)*	0.80	[0.48, 1.22]	.327	1
SES	-0.04 *(0.18)*	0.96	[0.49, 1.23]	.819	1
Education	0.62 *(0.29)*	1.86	[1.11, 3.51]	**.034***	.647
Sexual Orientation	-0.01 *(0.22)*	0.99	[0.61, 1.49]	.977	1
**Goodness of Fit**					
Information criteria	AIC = 261.2;	BIC = 349.3;	R^2 = 13.9 %		
Likelihood-ratio test	χ^2 = 27.8;	*df* = 20;	*p* = .114		

Note. [1] Coefficients *z*-standardized. One-sided *z*-tests were used for the BSRI (m), MRNI (including all its subscales), and GRCS (including all its subscales). *β* = estimate, *SE* = standard error, *CI = confidence interval*, *p* = *p*-value, *corr.* = corrected for multiple testing using the Holm-Bonferroni method, R^2 = Nagelkerke pseudo *R*-squared.

[a] PHQ-9 = Patient Health Questionnaire – 9; [b] MDRS-22 = Male Depression Risk Scale – 22; [c] BSRI (m) = Bem Sex-Role Inventory (masculinity subscale); [d] MRNI = Male Role Norms Inventory (subscales: NT = Negativity toward Sexual Minorities; RE = Restrictive Emotionality; SR = Self-reliance through Mechanical Skills; AF = Avoidance of Femininity; IS = Importance of Sex; D = Dominance; T = Toughness); [e] GRCS = Gender Role Conflict Scale (subscales: SPC = Success, Power, Competition; RE = Restrictive Emotionality; RABBM = Restrictive Affectionate Behavior Between Men; CBWFR = Conflicts Between Work and Leisure - Family Relations); [f] FCV-19S = Fear of COVID-19 Scale; [g] CPSS = COVID-19 Pandemic Stress Scale.

* = *p* < .05

Table S5. *Results of Univariate, Binary Logistic Regression Analyses predicting COVID-19 Infection*

**Variable**	**β[1] *(SE)*[1]**	**OR[1]**	**95% CI**	***p***	***p (corr.)***
**PHQ-9[a]**	-0.22 *(0.18)*	0.80	[0.56, 1.14]	.226	1
**MDRS-22[b]**	0.23 *(0.16)*	1.25	[0.90, 1.69]	.150	1
**BSRI (m)[c]**	0.22 *(0.18)*	1.24	[0.92, ____[	.116	1
**MRNI[d] (total)**	0.02 *(0.01)*	1.02	[1.00, ____[	**.008****	.158
(NT)	0.29 *(0.13)*	1.33	[1.06, ____[	**.014***	.263
(RE)	0.33 *(0.16)*	1.39	[1.06, ____[	**.018***	.313
(SR)	0.24 *(0.18)*	1.27	[0.94, ____[	.096	.961
(AF)	0.27 *(0.15)*	1.31	[1.01, ____[	**.035***	.567
(IS)	0.25 *(0.15)*	1.28	[0.99, ____[	**.045***	.625
(D)	0.38 *(0.13)*	1.46	[1.16, ____[	**.002****	**.043***
(T)	0.29 *(0.17)*	1.33	[1.00, ____[	**.049***	.647
**GRCS[e] (total)**	0.03 *(0.01)*	1.03	[1.01, ____[	**.013***	.261
(SPC)	0.38 *(0.18)*	1.47	[1.09, ____[	**.017***	.307
(RE)	0.25 *(0.18)*	1.28	[0.96, ____[	.077	.918
(RABBM)	0.23 *(0.16)*	1.26	[0.96, ____[	.076	.918
(CBWFR)	0.14 *(0.18)*	1.15	[0.86, ____[	.210	1
**FCV-19S[f]**	-0.26 *(0.20)*	0.77	[0.51, 1.12]	.201	1
**CPSS[g]**	-0.26 *(0.17)*	0.77	[0.55, 1.08]	.131	1
**Covariates**					
Age	0.02 *(0.17)*	1.02	[0.69, 1.39]	.924	1
SES	0.01 *(0.16)*	1.01	[0.58, 1.27]	.932	1
Education	0.33 *(0.16)*	1.39	[1.05, 1.95]	**.037***	.567
Sexual Orientation	-0.14 *(0.20)*	0.87	[0.56, 1.23]	.480	1

Note. [1] Coefficients *z*-standardized. One-sided *z*-tests were used for the BSRI (m), MRNI (including all its subscales), and GRCS (including all its subscales). *β* = estimate, *SE* = standard error, *CI = confidence interval*, *p* = *p*-value, *corr.* = corrected for multiple testing using the Holm-Bonferroni method.

[a] PHQ-9 = Patient Health Questionnaire – 9; [b] MDRS-22 = Male Depression Risk Scale – 22; [c] BSRI (m) = Bem Sex-Role Inventory (masculinity subscale); [d] MRNI = Male Role Norms Inventory (subscales: NT = Negativity toward Sexual Minorities; RE = Restrictive Emotionality; SR = Self-reliance through Mechanical Skills; AF = Avoidance of Femininity; IS = Importance of Sex; D = Dominance; T = Toughness); [e] GRCS = Gender Role Conflict Scale (subscales: SPC = Success, Power, Competition; RE = Restrictive Emotionality; RABBM = Restrictive Affectionate Behavior Between Men; CBWFR = Conflicts Between Work and Leisure - Family Relations); [f] FCV-19S = Fear of COVID-19 Scale; [g] CPSS = COVID-19 Pandemic Stress Scale.

* = $p < .05$, ** = $p < .01$

Table S6. *Variance Inflation Factors (VIF) for the Logistic Regression Models*

Predictor	Model 1 (no subscales)	Model 2 (with subscales)
**PHQ-9[a]**	2.38	2.67
**MDRS-22[b]**	2.22	2.34
**BSRI (m)[c]**	1.47	1.66
**MRNI[d] (total)**	1.67	
(NT)		2.69
(RE)		3.10
(SR)		2.03
(AF)		3.64
(IS)		2.21
(D)		4.09
(T)		3.26
**GRCS[e] (total)**	1.93	
(SPC)		1.67
(RE)		1.53
(RABBM)		1.76
(CBWFR)		1.52
**FCV-19S[f]**	1.59	1.64
**CPSS[g]**	1.67	1.79
**Covariates**		
Age	1.18	1.40
SES	1.04	1.07
Education	1.08	1.10
Sexual Orientation	1.12	1.17

[a] PHQ-9 = Patient Health Questionnaire – 9; [b] MDRS-22 = Male Depression Risk Scale – 22; [c] BSRI (m) = Bem Sex-Role Inventory (masculinity subscale); [d] MRNI = Male Role Norms Inventory (subscales: NT = Negativity toward Sexual Minorities; RE = Restrictive Emotionality; SR = Self-reliance through Mechanical Skills; AF = Avoidance of Femininity; IS = Importance of Sex; D = Dominance; T = Toughness); [e] GRCS = Gender Role Conflict Scale (subscales: SPC = Success, Power, Competition; RE = Restrictive Emotionality; RABBM = Restrictive Affectionate Behavior Between Men; CBWFR = Conflicts Between Work and Leisure - Family Relations); [f] FCV-19S = Fear of COVID-19 Scale; [g] CPSS = COVID-19 Pandemic Stress Scale.

S-Discussion: Discussion part on traditional masculinity and specific COVID-19-related symptoms

Possible group differences between men with high and low traditional masculinity regarding expression of COVID-19 symptoms in men who contracted COVID-19 were investigated. However, the exploratory analysis included only a subsample of 34 men reporting to have contracted COVID-19. As can be seen in Figure 4A, men with high traditional masculinity operationalized as high BSRI scores exhibited less general (i.e. pain symptoms, headache, joint pain, muscle pain, peripheral neuropathy) pain symptomatology (47.4%) as compared to men with low traditional masculinity (80.0%) and lower PHQ-9 scores as compared to men with low traditional masculinity. This is line with previous research showing studies using the BSRI-M to report higher masculine gender orientation to be positively associated with constructs such as self-esteem and self-confidence and negatively associated with depression and anxiety symptoms (Johnson & McCoy, 2000; Long, 1986; O'Heron & Orlofsky, 1990). Nevertheless, although non-significant due to the very small sample size, a clear picture is observable, that in general men with higher BSRI-M scores report overall less COVID-19 symptomatology. With regard to the MRNI-SF and the GRCS-SF this is not the case suggesting the masculinity measures based on the gender role strain paradigm in contrast to the gender role identity paradigm (BSRI-M) reveal more complex associations and suggest to examine specific subscales in order to identify, which specific role norms or gender role conflicts are related to symptom expression.
